# Supplementary material for: The effect of changing diagnostic method from culture to PCR on the number of episodes of human campylobacteriosis in Denmark: a retrospective study (2015–2022)
Source: Microbiol Spectr. 2023 Dec 8;12(1):e03418-23. doi: 10.1128/spectrum.03418-23 (PMC10783023; doi:10.1128/spectrum.03418-23)
Supplement: Supplemental material — Supplemental results: summary statistics of the base model, autocorrelation of residuals, and sensitivity analysis. [file spectrum.03418-23-s0001.pdf]

Supplementary materials

The effect of changing diagnostic method from culture to PCR on the number of episodes of human campylobacteriosis in Denmark: a retrospective study (2015-2022)

Author names and affiliations

Guido Benedetti[a], Christian Holm Hansen[a], Anna Tølbøll Svendsen[a,b,c], Katrine Grimstrup Joensen[d], Gitte Sørensen[d], Anne Line Engsbro[e,f], Mia Torpdahl[d], Eva Møller Nielsen[d], Steen Ethelberg[a,g]

[a] Department of Infectious Disease Epidemiology and Prevention, Statens Serum Institut, Artillerivej 5, 2300 Copenhagen, Denmark

[b] Department of Medicine, Zealand University Hospital, Lykkebækvej 1, 4600 Køge, Denmark

[c] Department of Clinical Medicine, University of Copenhagen, Blegdamsvej 3B, 2200 Copenhagen, Denmark

[d] Department of Bacteria, Parasites and Fungi, Statens Serum Institut, Artillerivej 5, 2300 Copenhagen, Denmark

[e] Department of Clinical Microbiology, Zealand University Hospital, Ingemannsvej 46, 4200 Slagelse, Denmark

[f] Hvidovre University Hospital, Kettegård Allé 30, 2650 Hvidovre, Denmark

[g] Department of Public Health, Global Health Section, University of Copenhagen, Øster Farimagsgade 5, 1353 Copenhagen, Denmark

Results – Summary statistics of the base model

Call:

glm.nb(formula = count_week ~

seq_week + sin52 + cos52 + sin26 + cos26 + ox_covid + pcr + kma,

data = d_base, init.theta = 11.12161928, link = log)

Deviance Residuals:

Min	*1Q*	*Median*	*3Q*	*Max*
-41.721	-0.7747	-0.1025	0.5361	106.014

Coefficients:

	Estimate	*Std. Error*	*z value*	*Pr(>|z|)*	
(Intercept)	22.708.230	0.0384753	59.020	< 2e-16	***
seq_week	0.0001163	0.0001217	0.955	0.33941	
sin52	-0.5973987	0.0130515	-45.773	< 2e-16	***
cos52	-0.3623618	0.0125268	-28.927	< 2e-16	***
sin26	0.1365809	0.0123888	11.025	< 2e-16	***
cos26	0.0344867	0.0124029	2.781	0.00543	**
stringency_covid	-0.0070249	0.0005010	-14.022	< 2e-16	***
pcr	0.3590117	0.0342453	10.484	< 2e-16	***
kma_B	0.5726841	0.0449023	12.754	< 2e-16	***
kma_C	-12.191.404	0.0511990	-23.812	< 2e-16	***
kma_D	-0.6303622	0.0385313	-16.360	< 2e-16	***
kma_E	-0.0407790	0.0358689	-1.137	0.25558	
kma_F	-11.172.492	0.0442172	-25.267	< 2e-16	***
kma_G	-12.433.624	0.0454491	-27.357	< 2e-16	***
kma_H	-0.3304066	0.0387466	-8.527	< 2e-16	***
kma_I	0.1156141	0.0389979	2.965	0.00303	**

Signif. codes: 0 ‘***’ 0.001 ‘**’ 0.01 ‘*’ 0.05 ‘.’ 0.1 ‘ ’ 1

(Dispersion parameter for Negative Binomial(11.1216) family taken to be 1)

Null deviance: 13650.8 on 3223 degrees of freedom

Residual deviance: 3390.4 on 3208 degrees of freedom

AIC: 16444

Number of Fisher Scoring iterations: 1

Theta: 11.122

Std. Err.: 0.651

2 x log-likelihood: -16410.081

Results – Autocorrelation of residuals

As one of two approaches to address autocorrelation of residuals, the base model was run on one thousand random draws of 806 weekly observations each (a quarter of the weekly observations in the base dataset) in a Monte Carlo simulation. This allowed to empirically measure the PCR effect as the mean estimate of the simulation and its 95%CI as the 2.5th and 97.5th percentiles of the estimates of the simulation. The graph below describes the number of random draws in the simulation (vertical axis) and the simulated one thousand estimates of the PCR effect (horizontal axis). The box summarizes the empirically measured PCR effect and its 95%CI.

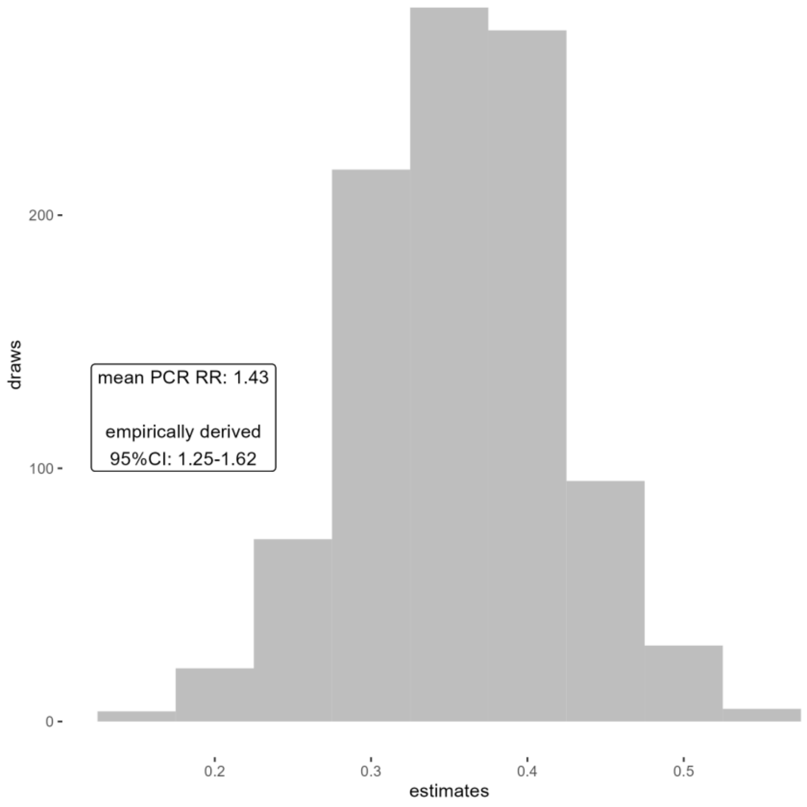

Results – Sensitivity analysis

Model	Description of the applied method	PCR-effect
Base-model	*See manuscript*	1.43 [1.34-1.53]
Model-2	The base model was run on the base dataset with an interaction term between the explanatory variables DCM and primary diagnostic method, thereby estimating separate PCR effects by each DCM	
DCM-A		1.62 [1.42-1.85]
DCM-D		1.31 [1.15-1.49]
DCM-E		1.69 [1.50-1.90]
DCM-I		1.30 [1.19-1.42]
Model-3	The base model was run on data from 2018 to 2021 only, a period when TBR was based on information from MiBa and data is complete for all DCMs	1.37 [1.21-1.56]
Model-4	The base model was run on the subset of data from the 4 DCMs that changed the primary diagnostic method during the study period	1.32 [1.22-1.42]
Model-5	The base model was run on the base dataset including the explanatory variable DCM as a random rather than a fixed effect	1.44 [1.36-1.52]
Model 6-to-9	The base model was run individually for each of the 4 DCMs that changed the primary diagnostic method during the study period	
DCM-A		1.39 [1.05-1.82]
DCM-D		1.31 [1.11-1.55]
DCM-E		1.49 [1.28-1.74]
DCM-I		1.31 [1.15-1.50]
Model-10	The base model was run on the base dataset excluding episodes of human campylobacteriosis with a known travel history in relation to the infection	1.34 [1.25-1.44]

Model-11	The base model was run on the base dataset excluding the 2% highest weekly observations ever occurring per DCM as such observations possibly reflect the occurrence of clustered outbreaks	1.39 [1.31-1.49]
Model-12	The base model was run on the base dataset after defyining the missing observations from DCM-A and DCM-B before 2018. To do this, first we calculated the weekly ratio of the reported campylobacteriosis episodes from DCM-A and DCM-B in the period 2018-2022. Secondly, we defined the weekly number of campylobacteriosis episodes for the two DCMs before 2018 by randomly selecting (with replacement) one of the calculated ratios and applying it to the available cumulated weekly number of episodes. In this way, we defined a weekly value for the two DCMs in the period when their observations could not be distinguished in TBR	1.37 [1.29-1.46]

DCM: Department of Clinical Microbiology; PCR: Polymerase Chain Reaction; TBR: Register of Enteric Pathogens; MiBa: Danish Microbiology Database
